# Supplementary material for: The interactome of intact mitochondria by cross-linking mass spectrometry provides evidence for coexisting respiratory supercomplexes
Source: Mol Cell Proteomics. 2017 Dec 8;17(2):216–32. doi: 10.1074/mcp.RA117.000470 (PMC5795388; doi:10.1074/mcp.RA117.000470)
Supplement: Supplemental Data [file supp_RA117.000470_133922_0_supp_23978_fzffwf.zip › spectra_annotation/mito_DR_spectra_annotation/98-1-2-1-7-1.pdf]

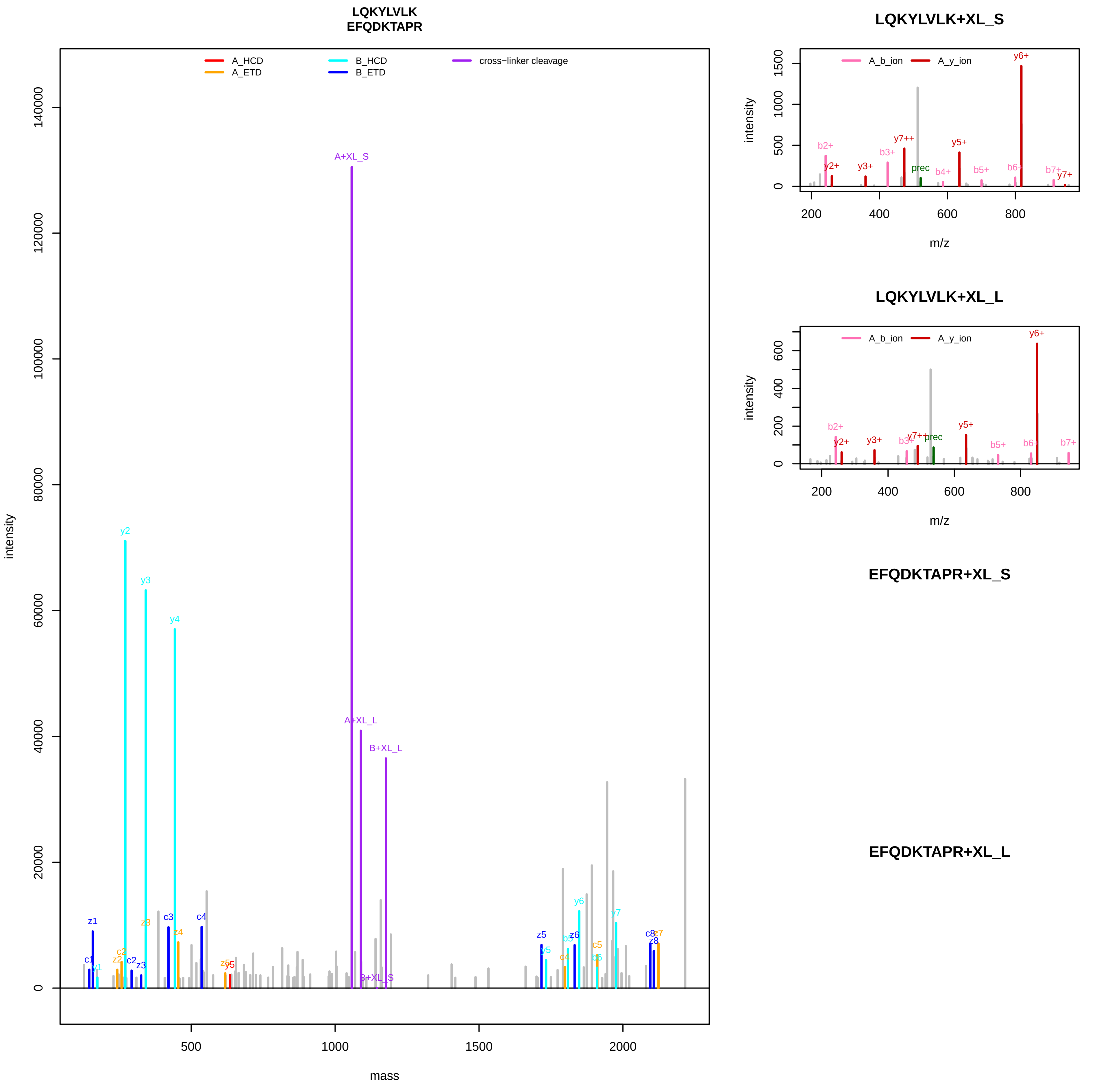
LQKYLVLK
EFQDKTAPR
A_HCD
A_ETD
B_HCD
B_ETD
cross−linker cleavage
A+XL_S
A+XL_L
B+XL_L
B+XL_S
y2
y3
y4
y5
y6
y7
c1
z1
y1
c2
z2
z3
c3
z4
c4
z5
c5
b5
b6
c8
z8
z7
intensity
mass
0
20000
40000
60000
80000
100000
120000
140000
500
1000
1500
2000
LQKYLVLK+XL_S
A_b_ion
A_y_ion
b2+
y2+
y3+
b3+
y7++
prec
b4+
y5+
b5+
b6+
y6+
b7+
y7+
intensity
m/z
LQKYLVLK+XL_L
EFQDKTAPR+XL_S
EFQDKTAPR+XL_L
